# 215b. Real-World Experience of Letermovir Use at an Academic Transplant Center

**DOI:** 10.1093/ofid/ofac492.293

**Published:** 2022-12-15

**Authors:** HaYoung Ryu, Christina Bowen, Joseph Bubalo, Diana Yu, James S Lewis

**Affiliations:** Oregon Health & Science University Hospital and Clinics, Portland, OR; Oregon Health and Science University, Portland, Oregon; Oregon Health and Science University, Portland, Oregon; Doernbecher Children's Hospital/OHSU Healthcare, Portland, Oregon; Oregon Health and Science University, Portland, Oregon

## Abstract

**Background:**

Letermovir (LMV) is indicated for cytomegalovirus (CMV) prophylaxis in seropositive adults after allogeneic hematopoietic stem cell transplants (alloSCT). LMV is well tolerated compared to its alternatives. Our institution restricts LMV use to high-risk patient groups to maximize the cost/benefit of LMV. Despite these restrictions, LMV was in our institution’s top 50 drug expenditures, prompting a formal evaluation of its use. We describe a real-world experience with LMV at a 576-bed academic transplant center.

**Methods:**

This was a single center, retrospective, descriptive study of LMV use at a transplant center. Any hospitalized patient who received ≥ 1 dose of LMV between August 2021 and January 2022 was eligible for analysis. Data collection included age, LMV administration data, length of stay, transplant status, Infectious Diseases (ID) consultation, documented rationale for non-criteria uses, and drug cost. The primary outcome was incidence of use not aligning with restriction criteria. The secondary outcome was institutional drug expenditure for non-criteria uses compared to approved uses.

**Results:**

Over 6 months, 388 doses of LMV were administered to 31 unique patients during 41 admissions. LMV use during 12/41 admissions (31.7%) fell outside the institutional criteria. ID consult occurred in 58.3% (7/12) of cases. 27/31 (87%) were alloSCT patients, including 1 pediatric patient. There was also 1 liver and 1 heart transplant recipient. Reasons for non-criteria use were: primary prophylaxis in the heart transplant patient to avoid ganciclovir toxicity and in 2 alloSCT patients without high risk for CMV, pre-emptive therapy due to CMV reactivation (n=2), and step-down therapy for CMV viremia (n=4). LMV was used for extended periods ( > 100 days from transplant) in 2 cases. Non-criteria use of LMV accounted for $20 414 in drug cost over 6 months, 29% of total expenditure.

**Conclusion:**

Our data suggest real world use of LMV often differs from the patient types enrolled in the phase 3 trials. Use of LMV as an alternative to available CMV therapies to avoid drug toxicities appears to drive much of this use. In addition, use outside of criteria was frequent and incurred a nontrivial cost. Modification of our criteria, such as requiring an ID consultation for off-label use, should be considered.

**Disclosures:**

**James S. Lewis, PharmD, FIDSA**, Cidara: Advisor/Consultant|Merck: Advisor/Consultant|SeLux Diagnostics: Advisor/Consultant.

